# Departrment of Surgery

**Published:** 1893-03

**Authors:** 


### DEPARTRMENT OF SURGERY.

Dr. Samuel E. Milliken, of the New York Polyclinic, recommends (*American Practitioner and News*) what he calls the "Hammock method" of applying plaster of Paris jacket to children for Potts disease. He says children have such horror of *being suspended* and resist so strongly as to prevent uniformity of pressure when released, that he was led to study this method ("which is not new") and to adopt it. He describes it as follows:

*Apparatus.*—By means of a strip of ordinary domestic four feet long and six or eight inches broad, stretched between the backs of two ordinary chairs, the child is placed either in the prone or supine position, with an assistant to hold its arms and another its lower extremities. The weight of each assistant is quite sufficient to prevent the tilting of the chairs, and the hammock is complete.

All the bony prominences, such as the spinous processes over the kyphosis and the crests of the ilia, are protected from pressure by one or two thicknesses of blanket before any plaster is applied. Each bandage is immersed in a basin of water until completely saturated, that no lumps may be present or delay entailed while being applied. The first roll should begin either at the pelvis or axillæ, and from four to six thicknesses applied uniformly, which, if allowed to harden without the patient being disturbed, will be quite sufficient support.

The minimum amount of plaster will insure the greatest comfort for the wearer of the jacket. The plaster having become hard, the strip of muslin may be cut off even with the plaster edges, or, by having been placed inside the seamless shirt, it can be entirely withdrawn.

The plaster is now cut out under the arms and over the thighs that too much pressure may not be exerted in the axillæ, and the patient be permitted to sit without discomfort.

The jacket may be worn without discomfort for from one to six months, depending on the weather and the absence or presence of foreign bodies, such as coins, bottons and the like, which are often a great source of annoyance in producing excoriations.

*Conclusion.*—The advantages of the hammock over the suspension method are:

1. Cheapness.
2. Practicability.
3. Efficiency, by enabling us to get a perfect cast of the body without resistance from the child.

